# Secretagogin Mediates the Regulatory Effect of Electroacupuncture on Hypothalamic-Pituitary-Adrenal Axis Dysfunction in Surgical Trauma

**DOI:** 10.1155/2021/8881136

**Published:** 2021-02-04

**Authors:** Mizhen Zhang, Jingxian Sun, Yu Wang, Zhanzhuang Tian

**Affiliations:** Department of Integrative Medicine and Neurobiology, School of Basic Medical Sciences, State Key Laboratory of Medical Neurobiology, Institutes of Brain Science, Institute of Acupuncture Research, WHO Collaborating Center for Traditional Medicine, Academy of Integrative Medicine, Fudan University, Shanghai, China

## Abstract

Electroacupuncture (EA) improves hypothalamic-pituitary-adrenal (HPA) axis disorder by reducing corticotropin-releasing hormone (CRH) synthesis and release in the paraventricular nucleus (PVN). However, the potential mechanism underlying CRH regulation remains unclear. Secretagogin (SCGN) is closely related to stress and is involved in regulating the release of CRH. We hypothesized that SCGN in the PVN might trigger the HPA system and be involved in EA-mediated modulation of HPA dysfunction caused by surgical trauma. Serum CRH and adrenocorticotropic hormone (ACTH) and plasma corticosterone (CORT) levels at 6 h and 24 h after hepatectomy were determined by radioimmunoassay. CRH and SCGN protein levels in the PVN were detected by western blot and immunofluorescence, and CRH and SCGN mRNA levels in the PVN were determined by means of real-time polymerase chain reaction (RT-PCR) and *in situ hybridization* (ISH). Our studies showed that serum CRH, ACTH, and CORT levels and PVN CRH expression were significantly increased at 6 h and 24 h after hepatectomy in the hepatectomy group compared with the control group, and those in the EA+hepatectomy group were decreased compared with those in the hepatectomy group. The protein and mRNA levels of SCGN in the PVN were also increased after hepatectomy, and their expression in the EA+hepatectomy group was decreased compared with that in the hepatectomy group. When SCGN expression in the PVN was functionally knocked down by a constructed CsCI virus, we found that SCGN knockdown decreased the serum CRH, ACTH, and CORT levels in the SCGN shRNA+hepatectomy group compared with the hepatectomy group, and it also attenuated CRH expression in the PVN. In summary, our findings illustrated that EA normalized HPA axis dysfunction after surgical trauma by decreasing the transcription and synthesis of SCGN.

## 1. Introduction

Surgery can improve health conditions while bringing about a series of adverse consequences, such as endocrine dysfunction [1], inflammation [2], immunosuppression [3], depression [4], and loss of memory [5], even with the rapid development of surgical techniques. Although a few treatments can relieve adverse reactions caused by surgery [6], it is still difficult to recover from the surgery-induced stress response.

The hypothalamic-pituitary-adrenal (HPA) axis, a crucial component of the stress response [7], plays an important role in maintaining homeostasis, and the paraventricular nucleus (PVN) is the initial and central part of the HPA axis. The corticotropin-releasing hormone (CRH) neurons of the PVN act as the “stress center” and synthesize and release CRH into hypophyseal portal vessels at the median eminence [8]. Adrenocorticotropic hormone (ACTH) is released from the anterior lobe of the pituitary, which is triggered by CRH, and ACTH can stimulate the adrenal cortex to secrete glucocorticoids (GCs) and then mobilize energy utilization by binding to glucocorticoid receptors [9]. Nevertheless, the mechanism of regulation of CRH synthesis and release in trauma is still obscure. It is necessary to investigate new targets for improving HPA axis dysfunction caused by surgical trauma.

Electroacupuncture (EA) has been applied for the management of perioperative complications and has the benefit of reducing duration of postoperative ileus after laparoscopic surgery for colorectal cancer to promote recovery [10], limiting neuroinflammation as well as postoperative cognitive function. EA under general anesthesia can improve postoperative recovery [11] and has been proven to have cardioprotective effect in rats of chest trauma [12]. In addition, previous studies from our lab also found that EA could normalize the hyperactivity of the HPA axis induced by hepatectomy through regulating the activity of hypothalamic neurons [13] and ameliorates immunosuppression and complications caused by surgical trauma [14]. Nevertheless, the mechanism by which EA alleviates HPA axis hyperfunction is still unclear.

Secretagogin (SCGN), a member of the EF-hand superfamily of Ca^2+^-binding proteins, is considered a Ca^2+^ sensor protein that regulates cellular activity [15] primarily in the neuroendocrine axis and the central nervous system, particularly the locus in the PVN [16]. SCGN can mediate CRH secretion, and SCGN knockdown might result in CRH accumulation in the PVN and a cascade leading to blunting of ACTH and CORT production. However, the exact biological function of SCGN in CRH synthesis and release in the PVN during surgical trauma is not clear. Here, we sought to address whether EA alleviates the hyperactivity of the HPA axis caused by surgical trauma by regulating the synthesis and release of SCGN and to further clarify the mechanism by which EA improves HPA axis dysfunction in rats with acute surgical trauma.

## 2. Materials and Methods

### 2.1. Animals

Adult male (Sprague-Dawley) rats weighing 160 ± 10 g were purchased from the Shanghai SLAC Laboratory Animal Center (Chinese Academy of Sciences, Shanghai, China). All rats were housed under a 12 h light/dark cycle at 22°C with food and water *ad libitum*. All rats were acclimated to the experimental conditions for one week before the experiment. All experimental procedures involving the use of animals were conducted in accordance with NIH Guidelines (NIH Publication No. 8023, revised 1978) and approved by the Animal Care and Use Committee of Fudan University.

### 2.2. Surgery and Electroacupuncture

The rats were divided into control, hepatectomy, and EA+hepatectomy groups (*n* = 6 per group). All animals were adapted to a moderate restraint for 30 minutes per day for 3 days. The surgical trauma model used in this experiment was as described previously [13]. Briefly, after anesthetization by intraperitoneal injection of 2% pentobarbital sodium, rats were given a surgical incision, which was approximately 10 cm long from the cartilago ensiformis to the pubic symphysis along the linea alba. The abdominal cavity was fully opened, and 20% of the liver was removed from the right lobe. After 20% of the liver lobe was removed, the bleeding was stemmed by inserting a sterile dry cotton for 5 minutes. Finally, the abdominal cavity was sutured. All surgeries were performed under aseptic conditions and between 9:00 and 11:00 AM.

EA application was performed as described before. “Zu San Li” (ST36, located in the posterior lateral knee joint, approximately 0.5 cm below the capitulum fibulae) and “San Yin Jiao” (SP6, at the superior border of the medial malleolus, between the posterior border of the tibia and anterior border of the Achilles tendon) acupoints were selected in this experiment. Rats in the EA group received EA treatment with a 0.5-inch acupuncture needle that was connected to the output of a Han's Acupoint Nerve Stimulator (LH2000, Beijing, China). The stimulation lasted for 30 min (8:00–10:00 AM) at an intensity of 2 mA with strings of dense-sparse frequencies (2 Hz for 1.05 s and 15 Hz for 2.85 s, alternating).

### 2.3. Western Blot

For analysis of CRH and SCGN protein expression in the PVN, total PVN tissue was isolated from rat brains and homogenized by using radioimmunoprecipitation assay lysis buffer (P0013B, Beyotime, China). The supernatant was centrifuged at 12000 rpm for 30 min at 4°C, mixed with loading buffer (161-0747, Bio-Rad, Hercules, CA, USA), and denatured at 100°C for 15 min. Equal amounts of protein samples (30 *μ*g) were run on 10% SDS-PAGE and transferred onto PVDF membranes. The membrane was blocked for 2 h at room temperature in PBS containing 5% low-fat milk. Then, the membranes were incubated with primary antibodies against SCGN (14037S, 1 : 1000, Cell Signaling Technology, USA) and CRH (ab8901, 1 : 10000, Abcam, Cambridge, UK) overnight at 4°C, washed five times with Tris-buffered saline and Tween 20 (TBST), and incubated for 2 h at room temperature with horseradish peroxidase- (HRP-) conjugated goat anti-rabbit IgG (H+L). After the membranes were washed five more times, the blots were developed in ECL solution (Pierce, Rockford, US) for 3 min, and immunoreactivity bands were detected by using an ImageQuant LAS 4000 Mini System (GE Healthcare, Buckinghamshire, UK). Densitometry analysis was quantified by using Quantity One software (Bio-Rad, Hercules, CA, USA). *β*-Actin (20536-1-AP, 1 : 10000, Proteintech, Chicago, USA) was used as an internal reference.

### 2.4. Radioimmunoassay

After rats were placed under anesthesia, blood samples were collected into tubes and incubated at room temperature for 4 h. Then, these samples were centrifuged at 3000 rpm (4°C) for 30 min to isolate the plasma. The supernatant was transferred to new tubes and stored at −80°C. The serum CRH, ACTH, and CORT levels were detected by using kits from the Beijing Sino-UK Institute of Biological Technology. Briefly, all reagents and samples were prepared, and the reagents were added to the samples according to the instructions. After mixing, they were incubated at 4°C for 24 h, and then, the separating agent was added to each sample and incubated for 15 minutes at room temperature. After centrifugation at 3500 rpm for 15 minutes, the supernatant was discarded, and the radioactivity in the precipitate was counted. The sensitivity of the CRH kit was less than 0.16 ng/ml, and the intra- and interassay coefficients were less than 7.0% and 15%, respectively. The sensitivity of the ACTH kit was less than 0.4 pg/ml, and the intra- and interassay coefficients were less than 7.0% and 15%, respectively. The sensitivity of the CORT kit was 1 ng/mL, and the intra- and interassay coefficients of variation were 7.5% and 9.5%, respectively.

### 2.5. Real-Time Polymerase Chain Reaction

We performed quantitative real-time polymerase chain reaction (real-time PCR) as previously described [17]. Total RNA was isolated from PVN tissues by using TRIzol Reagent (15596026, Life Technologies, USA) and then reverse-transcribed to cDNA with a PrimeScript RT Reagent Kit (RR036A, Takara, Japan) according to the manufacturer's instructions. PCR was conducted with a SYBR Premix Ex Taq kit (RR420A, Takara, Japan) in an ABI 7300 Real-Time PCR System (Applied Biosystems, USA). The reaction volume included 10 *μ*l of SYBR Premix Ex Taq mix, 1.2 *μ*l of the primer mixture, 2 *μ*l of the cDNA template, and 6.8 *μ*l of ddH_2_O. The transcript level was normalized to that of the reference gene (GAPDH for endogenous genes) in the same sample. The mRNA primers were synthesized by Sangon Biotech Company (Sangon, Shanghai).

The following primer pairs were used: SCGN forward TGG ACA ACG CAC ACA GAC AA and SCGN reverse AAA GGC ATC CAG CTC TGT CTC; CRH forward CTC TCT GGA TCT CAC CTT CCAC and CRH reverse CTA AAT GCA GAA TCG TTT TGGC; and GAPDH forward GTA TGA CTC TAC CCA CGG CAA GT and GAPDH reverse TTC CCG TTG ATG ACC AGC TT. All experiments were run in triplicate, and relative mRNA levels were analyzed by means of the 2^−ΔΔ^Ct method and normalized to GAPDH.

### 2.6. Immunofluorescence Histochemistry

Rats were perfused with phosphate-buffered saline (0.1 M PBS, pH 7.4) followed by 4% paraformaldehyde (PFA) in 0.1 M PBS. When the brains were isolated, they were left in 4% PFA overnight and then immersed in 20% sucrose in 4% PFA. After the brains were immersed in 30% sucrose in 0.1 M PBS, sections were cut at a thickness of 30 *μ*m by using a freezing microtome (Leica 2000, Germany). These sections, which included the PVN area (interaural 7.56 mm, bregma -1.44 mm ~ interaural 6.96 mm, bregma -2.04 mm according to *The Rat Brain in Stereotaxic Coordinates*, *5th Ed.*), were washed five times for 5 min in 0.01 M PBS. Blocking was performed for 1 h at 37°C in Immunol Staining Blocking Buffer (P0102, Beyotime, China), and then, the sections were incubated overnight with a rabbit polyclonal anti-SCGN antibody (14037S, 1 : 500, Cell Signaling Technology, USA) at 4°C. After incubation, the sections were washed five times for 5 min each in 0.01 PBS and incubated with a goat anti-rabbit IgG (H+L) highly cross-adsorbed secondary antibody, Alexa Fluor 488 (A-11034, 1 : 1000, Thermo Fisher Scientific, USA), in 0.01 PBS for 2 h at room temperature. The slices were washed five times for five minutes each in 0.01 M PBS and mounted on glass slides with sealing liquid mounting media containing DAPI. All sections were observed under a fluorescence microscope (Leica, Germany).

### 2.7. In Situ Hybridization

Animals were anesthetized and perfused with 4% paraformaldehyde. When the brains were isolated, they were immediately shock-frozen in dry ice. They were cut at a thickness of 25 *μ*m by using a freezing microtome (Leica 2000, Germany). Specific sections were processed for *in situ hybridization* by using an Enhanced Sensitive ISH Detection Kit (MK1614, Boster Inc., Wuhan, China) according to the manufacturer's instructions. For quantification, the relative levels of mRNA expression were determined by computer-assisted optical densitometry.

### 2.8. Interference Adenovirus Injection

Rats were placed on an isothermal pad and mounted in a stereotaxic apparatus (68026, Reward, Shenzhen) after anesthetization by intraperitoneal injection of 2% sodium pentobarbital solution. After fixation with a cotton swab, the hair on the top of the head was shaved, and then, the surgical site was painted with a dilute iodine solution. A single incision was made on the top of the skull, and the surgical area was wiped with a surgical swab moistened with hydrogen peroxide until the skull was exposed. The location of the bregma was ascertained and served as the reference position for the injection sites. The PVN was located at the anteroposterior (AP) (+1.5) and lateral (±0.4) part. After holes were drilled in the skull, a total amount of 2 *μ*l of pDKD-CMV-mCherry-U6-shRNA adenovirus was injected into the dorsal-ventral (DV) -8.1 region, and the glass needle was held in place for at least 5 min. Ten minutes later, the same volume of pDKD-CMV-mCherry-U6-shRNA was injected into the other side. The rats were subjected to subsequent experiments 5 days after virus injection. The interfering virus used in this experiment was synthesized by OBiO Technology (Shanghai, China).

### 2.9. Statistical Analysis

All data were analyzed by using GraphPad Prism version 6.0 (CA, USA). Data are expressed as the mean ± SEM. Significance analysis was performed on raw data by one-way analysis of variance (ANOVA) with a significance level of *p* < 0.05 in two-tailed tests.

## 3. Results

### 3.1. EA Decreases the HPA Axis Hyperactivity Induced by Hepatectomy

**Figure 1:**
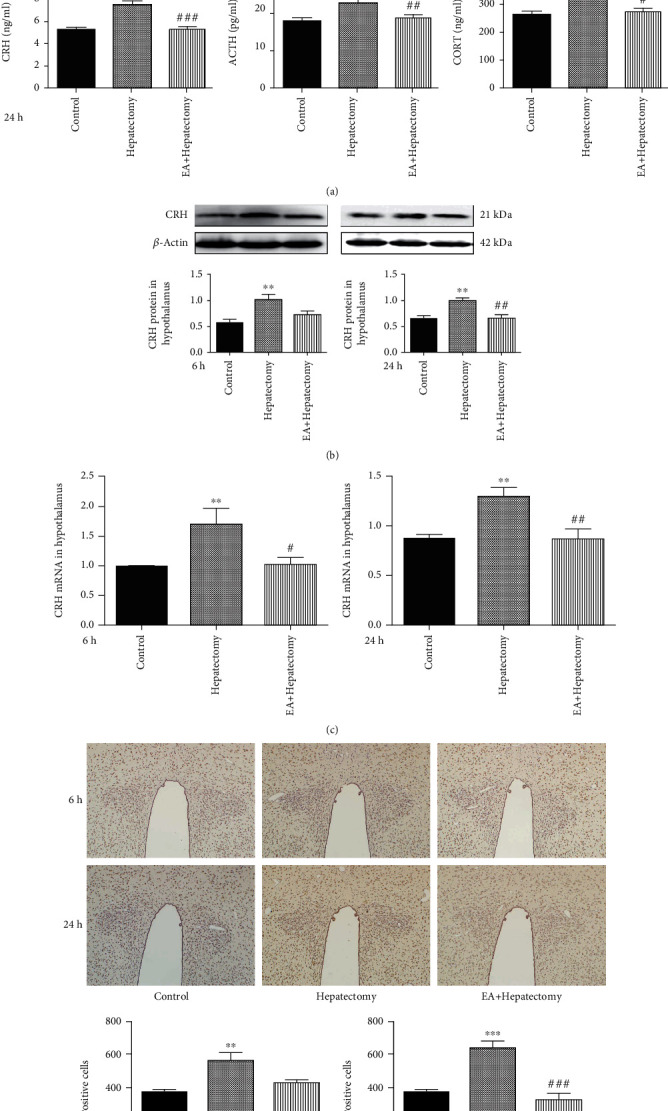
Peripheral serum CRH, ACTH, and CORT levels were detected by the radioimmunoassay after rats suffering surgical trauma and CRH expression in the PVN of rats. (a) The peripheral serum CRH, ACTH, and CORT levels in the control, hepatectomy, and EA+hepatectomy groups at 6 h and 24 h after hepatectomy, respectively. (b) Representative bands of CRH protein levels and quantification of CRH protein levels and (c) CRH mRNA levels at 6 h and 24 h in the control, hepatectomy, and EA+hepatectomy groups after hepatectomy. (d) Representative pictures of CRH mRNA-positive cells and quantification of CRH mRNA-positive cells in the control, hepatectomy, and EA+hepatectomy groups at 6 h and 24 h after hepatectomy. Data are presented as the mean ± SEM (*n* = 4). ∗ vs. control group (*p* < 0.05): ^∗∗^*p* < 0.01, ^∗∗∗^*p* < 0.001; **#** vs. hepatectomy group (*p* < 0.05): ^##^*p* < 0.01, ^###^*p* < 0.001.

To identify the regulation of EA in the hepatectomy rats, we detected the level of the serum CRH, ACTH, and CORT and found that at 6 h after hepatectomy, CRH, ACTH, and CORT levels were increased in the hepatectomy group in comparison with the control ones. However, they were decreased in the EA+hepatectomy group. And similar results were obtained at 24 h after hepatectomy (Figure 1(a)). Then, we observed the expression of CRH in the PVN. At 6 h after operation, by comparing with the control group, the CRH protein level in PVN was upregulated in the hepatectomy group, and in comparison with the hepatectomy group, CRH protein level was downregulated in the EA+hepatectomy group, but there was no statistical significance. At 24 h after operation, the PVN CRH protein level was increased in the hepatectomy group compared with the control ones and that was decreased in the EA+hepatectomy group compared with the hepatectomy ones (Figure 1(b)). At 6 h and 24 h after operation, the expression of CRH mRNA in PVN was increased in the hepatectomy group compared with the control ones, and the CRH mRNA level in the EA+hepatectomy group was decreased compared with that of the hepatectomy ones (Figure 1(c)). The number of CRH mRNA-positive cells was increased in the hepatectomy group compared with the control ones, and they were decreased in the EA+hepatectomy group compared with the hepatectomy ones (Figure 1(d)). Taken together, these results showed that EA can control the expression of CRH and reduce the excessive action of the HPA axis.

### 3.2. EA Decreases the Expression of SCGN in PVN in Rats after Hepatectomy

**Figure 2:**
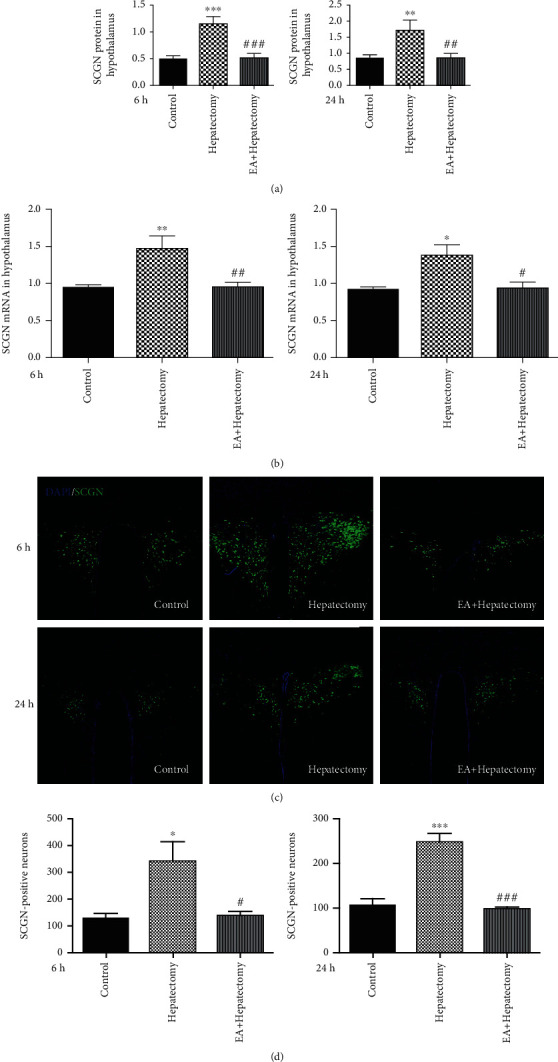
SCGN expression in the PVN of rats. (a) Representative bands of SCGN protein levels and quantification of SCGN protein levels and (b) SCGN mRNA levels at 6 h and 24 h in the control, hepatectomy, and EA+hepatectomy groups after hepatectomy. (c) Immunofluorescence staining of SCGN in the PVN among the control, hepatectomy, and EA+hepatectomy groups at 6 h and 24 h after surgery. Scale bar = 100 *μ*m. (d) Quantification of SCGN-positive neurons in the control, hepatectomy, and EA+hepatectomy groups at 6 h and 24 h after hepatectomy. Data are presented as the mean ± SEM (*n* = 4). ∗ vs. control group (*p* < 0.05): ^∗∗^*p* < 0.01, ^∗∗∗^*p* < 0.001; **#** vs. hepatectomy group (*p* < 0.05): ^##^*p* < 0.01, ^###^*p* < 0.001.

The abundant expression of SCGN in the PVN, which is related to CRH secretion, affected the HPA axis. Therefore, we want to explore the impact of EA on it; we examined the changes of SCGN expression after hepatectomy. At 6 h and 24 h after operation, the SCGN protein level in the PVN was increased in the hepatectomy group compared with the control ones, and the SCGN protein level in the EA+hepatectomy group was decreased compared with that in the hepatectomy ones (Figure 2(a)). And the expression of SCGN mRNA was increased in the hepatectomy group compared with the control ones, while it was decreased in the EA+hepatectomy group (Figure 2(b)). We also observed that the number of SCGN-positive neurons in the PVN had a similar trend (Figures 2(c) and 2(d)). These data confirmed that EA can regulate SCGN expression directly.

### 3.3. Functional Knockdown of the SCGN Expression in PVN by the Constructed CsCI Virus

**Figure 3:**
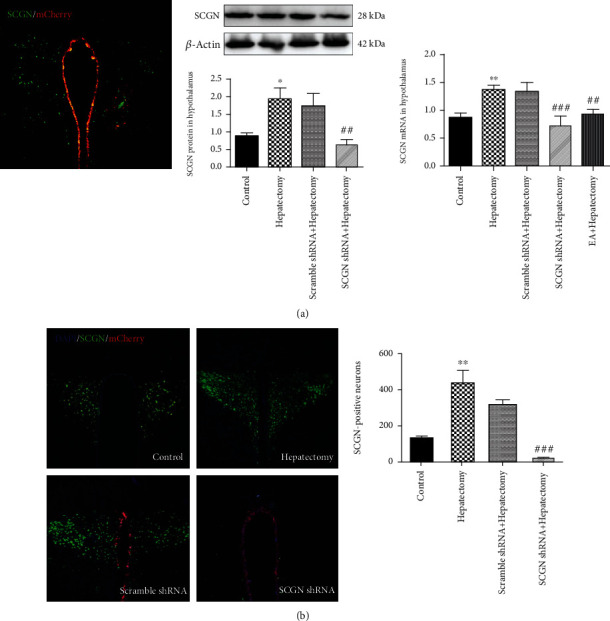
Virus-mediated shRNA knockdown of SCGN in the neurons of PVN in rats and the change of SCGN expression. (a) The CMV viral vector and the locations of the CMV injection sites in rat brain and SCGN levels in the control, hepatectomy, scramble shRNA+hepatectomy, and SCGN shRNA+hepatectomy groups at 24 h after hepatectomy. (b) Immunofluorescence staining of SCGN, GFP, and DAPI in the PVN and quantification of SCGN-positive neurons among the control, hepatectomy, scramble shRNA+hepatectomy, SCGN shRNA+hepatectomy, and EA+hepatectomy groups after surgery. Scale bar = 100 *μ*m. All data are shown as the mean ± SEM (*n* = 4). ∗ vs. control group (*p* < 0.05): ^∗∗^*p* < 0.01; **#** vs. hepatectomy group (*p* < 0.05): ^##^*p* < 0.01, ^###^*p* < 0.001.

To further probe the role of SCGN in the EA-mediated modulation of HPA axis dysfunction caused by surgical trauma, an adenovirus was constructed which could downregulate SCGN expression in the PVN. At 6 days after adenovirus injection, the red fluorescence of the mCherry carried by the adenovirus was detected in the PVN. The SCGN protein levels in the PVN of the SCGN shRNA+hepatectomy group were decreased compared with those in the hepatectomy ones, while the SCGN protein levels in the scramble shRNA+hepatectomy group were not different compared with those in the hepatectomy group. The SCGN mRNA levels in the PVN in the SCGN shRNA+hepatectomy group were downregulated compared with those in the hepatectomy group. However, there was no difference between the scramble shRNA+hepatectomy group and the hepatectomy group (Figure 3(a)). The number of SCGN-positive neurons in the PVN was decreased in the SCGN shRNA+hepatectomy group compared with the hepatectomy group (Figure 3(b)). These data indicated the excellent efficiency of functional knockdown of SCGN expression in the PVN in rats, and the role of SCGN in surgical trauma-induced HPA axis hyperfunction was further observed.

### 3.4. Knockdown of SCGN in PVN Modulates the Hyperactivity of the HPA Axis in Surgical Trauma

**Figure 4:**
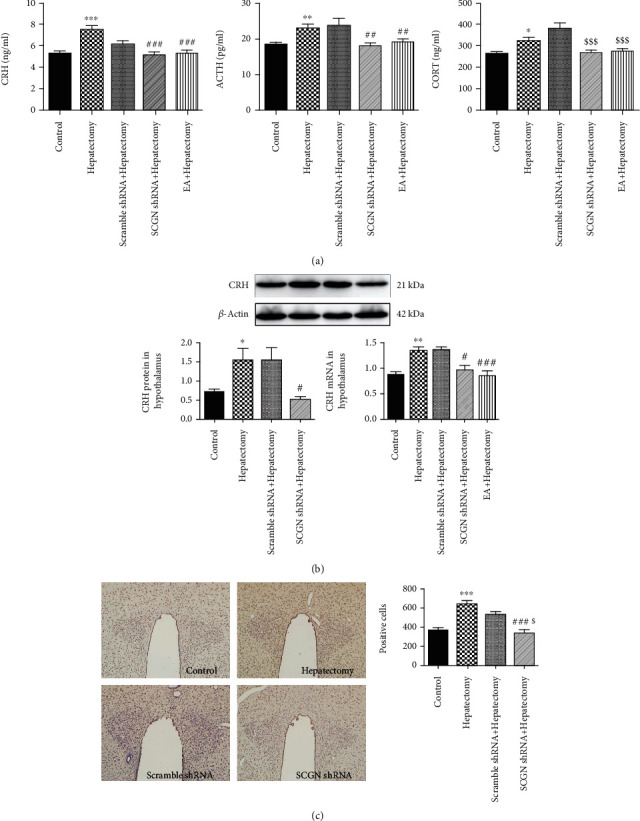
(a) Peripheral serum CRH, ACTH, and CORT levels were detected by the radioimmunoassay after rats suffering surgical trauma. (b) Representative bands and quantification of CRH protein level and CRH mRNA expression. (c) CRH-positive cells and quantification in the control, hepatectomy, scramble shRNA+hepatectomy, and SCGN shRNA+hepatectomy at 24 h after hepatectomy. Data are represented as the mean ± SEM (*n* = 6). ∗ vs. control group (*p* < 0.05): ^∗∗^*p* < 0.01, ^∗∗∗^*p* < 0.001; **#** vs. hepatectomy group (*p* < 0.05): ^##^*p* < 0.01, ^###^*p* < 0.001; $ vs. scramble shRNA+hepatectomy group (*p* < 0.05): ^$$^*p* < 0.01, ^$$$^*p* < 0.001.

When the SCGN is functionally knocked down in PVN, the role of SCGN in surgical trauma-induced HPA axis hyperfunction was observed. We found that the serum CRH, ACTH, and CORT levels in surgical trauma rats were changed after SCGN knockdown. Compared with the hepatectomy group, the CRH and ACTH levels were decreased in the SCGN shRNA+hepatectomy group; the CORT level in the SCGN shRNA+hepatectomy group was downregulated compared with that in the scramble shRNA+hepatectomy group (Figure 4(a)). We finally examined the CRH expression after SCGN knockdown. The CRH protein level was downregulated in the SCGN shRNA+hepatectomy group compared with the hepatectomy group. And there was no difference in CRH protein level between the scramble shRNA+hepatectomy and the hepatectomy group. Compared with the hepatectomy group, CRH mRNA levels were decreased in the SCGH shRNA+hepatectomy and EA+hepatectomy group. And there was no difference in CRH mRNA expression between the scramble shRNA+hepatectomy and the hepatectomy group (Figure 4(b)). The number of CRH-positive cells in the PVN was increased in the hepatectomy group compared with the control ones. However, when compared with the hepatectomy group, the number of CRH-positive cells in the PVN was decreased in the SCGN shRNA+hepatectomy group. But there was no difference in the number of SCGN-positive cells between the scramble shRNA+hepatectomy and the hepatectomy group (Figure 4(c)). Therefore, EA can mediate the SCGN expression to control the CRH section.

## 4. Discussion

Our previous study has demonstrated that surgical trauma might induce HPA axis hyperactivity [18]. Therefore, we wanted to strengthen the mechanism of EA on HPA axis dysfunction in surgical trauma. In this research, by observing the changes of the expression of SCGN and CRH in the PVN and the regulation of their expression by EA, we demonstrate that the role of SCGN mediates the regulatory effect of EA on HPA axis dysfunction in surgical trauma and provide a new target for the study of the mechanism of EA.

Glucocorticoids (GCs) play an important role in stress response by binding to their receptors [19]. A large amount of GCs are produced by the adrenal cortex during stress and negatively regulate the activity of hypothalamic CRH neurons and pituitary ACTH cells [20]. GCs also negatively regulate the mRNA expression of the ACTH precursor-POMC [21]. CORT, as the main GCs, participates in the regulation of stress response by binding to the corresponding receptors.

The changes in body fluids, electrolytes, and blood pressure caused by surgery are regulated by subfornical organ (SFO) neurons that excite the PVN neurons, and subsequently, CRH transcription in the PVN is significantly increased [22]. Therefore, CRH neurons in the PVN might directly affect the function of the HPA axis. Taking this into account, the CRH mRNA and protein levels in the PVN as well as the serum CRH, ACTH, and CORT levels were detected in our experiments. We found that the PVN CRH was increased after surgery, while EA downregulated its expression. EA also decreased the serum ACTH and CORT levels induced by surgery. These results strongly demonstrated that EA might regulate the HPA axis hyperfunction by affecting CRH expression in PVN.

Recently, a few studies have shown that SCGN is instrumental for CRH release in the median eminence and stress responsiveness. A cohort of parvocellular cells was shown to be interspersed with magnocellular PVN neurons expressing SCGN. When SCGN was conditionally knocked down, CRH accumulated in the PVN, and ACTH and CORT levels were significantly decreased during acute stress [16]. There has been more research on the involvement of SCGN in CRH release; however, there have been fewer studies about how SCGN regulates CRH synthesis. Thus, in our study, we first found that SCGN in the PVN was notably increased in surgical trauma rats and that SCGN also downregulated the synthesis of CRH in the PVN. EA treatment could significantly decrease the SCGN expression in PVN. These results indicated that SCGN is involved in the HPA axis hyperfunction and participated in EA-mediated modulation of HPA axis dysfunction caused by surgical trauma. However, the specific mechanism by which EA regulates SCGN expression requires further research.

Previous studies indicated that EA might alleviate the hyperactivity of the HPA axis after hepatectomy by modulating the expression of microRNAs and AVP and CRH receptors [23]. In addition, EA reduced central and peripheral inflammatory factor release in rats after surgical trauma by vagal activation [24]. A previous study indicated that EA at ST36 had an analgesic effect by regulating the expression of 5-HT1A and 5-HT3 receptors [25]. Additionally, EA at SP6 alleviated labor pain [26]. Our previous studies also showed that pre-EA reduced pain after laparotomy in rats [13]. EA enhanced 5-HT/5-HT1AR expression in the hippocampus to ameliorate chronic stress-induced depression-like behavior and stabilize the HPA axis [27]. In this study, we demonstrated that SCGN might be involved in the EA-mediated regulation of PVN CRH expression and reduction of the stress response.

In summary, our study showed that surgical trauma increased PVN CRH expression, which results in HPA axis hyperactivation. SCGN regulated the synthesis and secretion of CRH to participate in the acute stress induced by surgical trauma. EA alleviated the surgically induced HPA axis hyperfunction by downregulating SCGN expression. However, whether EA directly or indirectly affects the expression of SCGN still needs to be further probed.

## Data Availability

The figures used to support the findings of this study are included within the article.
